# Editorial of Special Issue “Rare Kidney Diseases: New Translational Research Approach to Improve Diagnosis and Therapy”

**DOI:** 10.3390/ijms21124244

**Published:** 2020-06-14

**Authors:** Gianluigi Zaza, Giovanni Gambaro

**Affiliations:** Renal Unit, Department of Medicine, University-Hospital of Verona, 37126 Verona, Italy; giovanni.gambaro@univr.it

In this Special Issue entitled “Rare Kidney Diseases: New Translational Research Approach to Improve Diagnosis and Therapy”, of the International Journal of Molecular Sciences, that includes original articles and reviews, authors have underlined the role of biomedical research in providing new insights into the pathologies of complex kidney diseases. 

Rare kidney diseases comprise a group of more than one hundred different life-threatening or chronically debilitating disorders affecting a small number of patients worldwide (approximately <1 in 2000 individuals in Europe and <200,000 in USA) [1] with different local and/or systemic clinical manifestations/complications. Most of them have a genetic basis, often affecting patients early in childhood, and are frequently progressive, disabling and life-threatening [2]. These features can have an overwhelming psychological impact on the families of children affected by these diseases.

However, although often perceived by the public opinion and media as a prime target of national health care systems, the research in this area has been, for many years, neglected in favor of more common diseases. Main reasons for the lack of interest in this field could be due to the small number of patients available and the consequent limited epidemiological data regarding many of these disorders.

Additionally, rare diseases can affect people in different ways. Even patients with the same disorder can exhibit very different signs and symptoms, or there may be many subtypes of the same condition. This diversity comprises a significant challenge to healthcare practitioners and scientists alike in terms of being able to gain sufficient experience for the most proper and timely definition, diagnosis and management.

A step forward in this field has been obtained by the advances in biotechnologies and ”omics” methodologies [3,4,5,6] that have led to the discovery of previously unrecognized disease-associated biological processes and identified new potential diagnostic biomarkers and drug targets. 

In this issue Bruschi et al. [7], by using a comprehensive comparative proteomic analysis (by mass spectrometry) of urinary microvesicles and exosomes, reported, for the first time, that several urinary proteins (some of them implicated in complement pathway regulation) may be able to clearly identify patients with medullary sponge kidney (MSK) disease, a rare kidney condition often associated with nephrocalcinosis/nephrolithiasis and cystic anomalies in the precalyceal ducts, from idiopathic calcium nephrolithiasis (ICN) as a control group.

The analysis of urinary extracellular vesicles is ideal because they can be easily obtained without invasive procedures, and they contain elevated levels of cell-specific proteins from every segment of the nephron, representing diverse cellular processes including metabolic, immunity-related and coagulation responses [8,9]. These intrinsic characteristics of extracellular vesicles could, therefore, provide a panel of informative marker proteins that not only allow the diagnosis and monitoring of MSK but could also provide insight into the underlying pathophysiological and biochemical processes.

Gianesello et al. [10] investigated allelic and locus heterogeneity in Dent disease (DD), an X-linked renal tubulopathy mainly caused by loss-of-function mutations in CLCN5 (DD1) and OCRL genes, and analyzed ClC-5, megalin, and cubilin expression in DD1 kidney biopsies. They further expanded the spectrum of CLCN5 mutations in DD by describing 23 novel mutations. In DD1 kidney biopsies, they showed that the loss of ClC-5 tubular expression caused defective megalin and cubilin trafficking. In DD3 patients (who have neither CLCN5 nor OCRL gene mutations) whole exome sequencing (WES) did not detect a new disease-causing gene. Instead, it revealed the concomitant presence of likely pathogenic variants in genes encoding proximal tubular endocytic apparatus components, suggesting that they may have had a role in determining the DD3 phenotype.

Furthermore, Muhle-Goll C et al. [11] investigated the accuracy of nuclear magnetic resonance (NMR)-based urine metabolomics for the diagnosis of acute kidney injury (AKI) in a pilot cohort study of neonates and children with established Kidney Disease: Improving Global Outcomes (KDIGO) AKI of heterogeneous etiology. They further explored if metabolomic fingerprints and biomarkers allow for a differentiation of specific AKI subtypes.

Multivariate analysis identified a panel of four metabolites that allowed diagnosis of AKI with an area under the receiver operating characteristics curve (AUC-ROC) of 0.95 (95% confidence interval 0.86–1.00). Especially urinary citrate levels were significantly reduced whereas leucine and valine levels were elevated. Metabolomic differentiation of AKI causes appeared promising but these results need to be validated in larger studies. 

De Rasmo D et al. [12] analyzed molecular aspects of nephropathic cystinosis, a rare inherited metabolic disease characterized by an impaired transport of the amino acid cystine out of lysosomes due to the reduced or absent function of the specific carrier cystinosin, which is encoded by the CTNS gene. 

In particular, authors investigated mitochondrial dynamics in CTNS^−/−^ conditionally immortalized proximal tubular epithelial cells (ciPTEC) carrying the classical homozygous 57-kb deletion with the intent of identifying new therapeutic targets and biomarkers for treatment follow-up.

Interestingly, their results clearly demonstrated that CTNS^−/−^ cells showed an overexpression of parkin associated with the deregulation of ubiquitination of mitofusin 2 and fission 1 proteins, an altered proteolytic processing of optic atrophy 1 (OPA1) and a decreased OPA1 oligomerization. According to molecular findings, the analysis of electron microscopy images showed a decrease in the mitochondrial cristae number and an increase in the cristae lumen and cristae junction width. Cysteamine treatment restored mitochondrial size, cristae number and lumen, but had no effect on cristae junction width, making tubular cells more susceptible to apoptotic stimuli. 

Based on their results, authors concluded that several cellular mediators of mitochondrial dynamics could be useful to develop new therapeutic interventions in this disease. This could be assessed by a future multicenter translational study.

The retrospective analysis of John C. Lieske group [13] investigated plasma oxalate (POx) as a potential predictor of end-stage kidney disease (ESKD) among primary hyperoxaluria (PH) patients. PH is a rare inherited autosomal recessive genetic disease caused by defects in genes that encode proteins important for glyoxylate metabolism [14]. 

Notably, results of this study demonstrated that in patients with PH, higher POx concentration was a risk factor for ESKD, particularly in advanced chronic kidney disease stages. 

Together with the aforementioned original articles, this issue also includes five literature reviews. 

Andrighetto et al. [15] reviewed the role of the complement cascade in a wide spectrum of rare renal diseases (including antibody-related glomerulopathies and non-antibody-mediated kidney diseases, such as C3 glomerular disease, atypical hemolytic uremic syndrome and focal segmental glomerulosclerosis) and the potential therapeutic effects of new selective complement-targeting drugs.

Schena FP group [16], describing recent research findings on C3 glomerulopathy, emphasized the importance of a multidisciplinary approach (that involves nephrologists, renal pathologists, molecular biologists and geneticists) to optimize the diagnosis and the treatment of this rare and neglected disease.

Fay J. Dickson and John A. Sayer [17] elegantly reviewed recent literature evidences regarding the employment of a novel precision-medicine approach to ensure patients affected by nephrocalcinosis and their families receive prompt diagnosis (that may slow down the progression to CKD), tailored treatment and accurate prognostic information (it is also useful to screen other family members).

Next, Sallustio et al. [18], based on recent literature evidences, have suggested a new vision of IgA Nephropathy, a primary glomerulonephritis that affects people mainly in the 2^nd^ and 3^rd^ decade of life. Whole-genome genomic studies revealed that this disorder is influenced by several environmental and behavioral factors that, if promptly corrected, may change the course of the disease.

Finally, Letavernier E et al., in an interesting narrative review [19], summarized recent discoveries concerning the pathophysiology of Pseudoxanthoma elasticum, a rare mendelian disease responsible for both cardiovascular and renal papillary calcifications, and discussed the potential implications of pyrophosphate deficiency as a promoter of vascular calcifications in kidney stone formers and in patients affected by CKD.

Overall, the 10 contributions have clearly shown that, in the future, molecular biology will certainly impact clinical decision making in nephrology and will become part of the day-to-day clinical practice.

## References

[1] Devuyst O., Knoers N.V., Remuzzi G., Schaefer F., Board of the working group for inherited kidney diseases of the European renal association and European dialysis and transplant association (2014). Rare inherited kidney diseases: Challenges, opportunities, and perspectives. Lancet.

[2] Bakurov I., Castelli M., Vanneschi L., Freitas M.J., Kaufmann P., Castillo P. (2019). Supporting medical decisions for treating rare diseases through genetic programming. Applications of Evolutionary Computation. EvoApplications 2019.

[3] Xie J., Liu L., Mladkova N., Li Y., Ren H., Wang W., Cui Z., Lin L., Hu X., Yu X. (2020). The genetic architecture of membranous nephropathy and its potential to improve non-invasive diagnosis. Nat. Commun..

[4] Sanna-Cherchi S., Khan K., Westland R., Krithivasan P., Fievet L., Rasouly H.M., Ionita-Laza I., Capone V.P., Fasel D.A., Kiryluk K. (2017). Exome-wide Association Study Identifies GREB1L Mutations in Congenital Kidney Malformations. Am. J. Hum. Genet..

[5] Fabris A., Bruschi M., Santucci L., Candiano G., Granata S., Gassa A.D., Antonucci N., Petretto A., Ghiggeri G.M., Gambaro G. (2017). Proteomic-based research strategy identified laminin subunit alpha 2 as a potential urinary-specific biomarker for the medullary sponge kidney disease. Kidney Int..

[6] Bruschi M., Granata S., Santucci L., Candiano G., Fabris A., Antonucci N., Petretto A., Bartolucci M., Del Zotto G., Antonini F. (2019). Proteomic Analysis of Urinary Microvesicles and Exosomes in Medullary Sponge Kidney Disease and Autosomal Dominant Polycystic Kidney Disease. Clin. J. Am. Soc. Nephrol..

[7] Bruschi M., Granata S., Candiano G., Fabris A., Petretto A., Ghiggeri G.M., Gambaro G., Zaza G. (2019). Proteomic analysis of urinary extracellular vesicles reveals a role for the complement system in medullary sponge kidney disease. Int. J. Mol. Sci..

[8] Pisitkun T., Shen R.-F., Knepper M.A. (2004). Identification and proteomic profiling of exosomes in human urine. Proc. Natl. Acad. Sci. USA.

[9] Moon P.-G., You S., Lee J., Hwang D., Baek M.-C. (2011). Urinary exosomes and proteomics. Mass Spectrom. Rev..

[10] Gianesello L., Ceol M., Bertoldi L., Terrin L., Priante G., Murer L., Peruzzi L., Giordano M., Paglialonga F., Cantaluppi V. (2020). Genetic analyses in dent disease and characterization of CLCN5 mutations in kidney biopsies. Int. J. Mol. Sci..

[11] Muhle-Goll C., Eisenmann P., Luy B., Kölker S., Tönshoff B., Fichtner A., Westhoff J. (2020). Urinary NMR profiling in pediatric acute kidney injury—A pilot study. Int. J. Mol. Sci..

[12] De Rasmo D., Signorile A., De Leo E., Polishchuk E., Ferretta A., Raso R., Russo S., Polishchuk R., Emma F., Bellomo F. (2020). Mitochondrial Dynamics of Proximal Tubular Epithelial Cells in Nephropathic Cystinosis. Int. J. Mol. Sci..

[13] Shah R.J., Vaughan L., Enders F., Milliner D., Lieske J.C. (2020). Plasma oxalate as a predictor of kidney function decline in a primary hyperoxaluria cohort. Int. J. Mol. Sci..

[14] Cochat P., Rumsby G. (2013). Primary hyperoxaluria. New Engl. J. Med..

[15] Andrighetto S., Leventhal J., Zaza G., Cravedi P. (2019). Complement and complement targeting therapies in glomerular diseases. Int. J. Mol. Sci..

[16] Schena F.P., Esposito P., Rossini M. (2020). A Narrative review on C3 glomerulopathy: A rare renal disease. Int. J. Mol. Sci..

[17] Dickson F., Sayer J.A. (2020). Nephrocalcinosis: A review of monogenic causes and insights they provide into this heterogeneous condition. Int. J. Mol. Sci..

[18] Sallustio F., Curci C., Di Leo V., Gallone A., Pesce F., Gesualdo L. (2010). A new vision of IgA nephropathy: The missing link. Int. J. Mol. Sci..

[19] Letavernier E., Bouderlique E., Zaworski J., Martin L., Daudon M. (2019). Pseudoxanthoma elasticum, kidney stones and pyrophosphate: From a rare disease to urolithiasis and vascular calcifications. Int. J. Mol. Sci..

